# Parents’ and carers’ attitudes to the use of digital technology and its role in the care of children with complex needs

**DOI:** 10.1177/03080226241233112

**Published:** 2024-03-19

**Authors:** Joanna Apps, Stephen Webb, Eve Hutton

**Affiliations:** 1 Department of Research Development, Faculty of Medicine, Health and Social Care, Canterbury Christ Church University, Canterbury, UK; 2 School of Allied and Public Health Professions, Faculty of Medicine, Health and Social Care, Canterbury Christ Church University, Canterbury, UK; 3 School of Allied and Public Health Professions, Faculty of Medicine, Health and Social Care, Canterbury Christ Church University, Canterbury, UK

**Keywords:** Parents and carers, rehabilitation therapies, digital technology, occupational therapy, children with complex needs

## Abstract

**Introduction:**

Parent/carers of disabled children want timely and personalized support. Research suggests that technology may address some limitations associated with traditional methods of communication with therapists (e.g. letter, telephone). This exploratory study examined United Kingdom (UK) parents and carers views on the use of digital technology (i.e. computers/phones) in supporting their child and the potential for its greater use in the care of children with complex needs.

**Methods:**

An online survey was distributed via special schools and support forums/networks. Questions explored use of and attitudes to digital technology in the care of children with complex needs. Descriptive statistical analyses and content analyses were undertaken on the data.

**Results:**

Respondents were 43 parents/carers whose children used rehabilitation services prior to the COVID-19 pandemic. The majority used digital technology frequently to support their child and saw the potential for greater use in rehabilitation services – provided this was not at the expense of in-person therapist contact.

**Conclusion:**

Parents and carers held positive views of digital technology as a tool to support their child and enhance rehabilitation services. Recommendations include regular service consultation on parental/child attitudes to digital service delivery and longitudinal studies to assess related health outcomes.

## Introduction

Smartphone use is now widespread (Ofcom, 2018) and interest in how to capitalize on this and the use of related mobile technology (e.g. tablets and laptops) to facilitate communication between patients and clinicians has increased (Shaw et al., 2020). For patients leading complex lives with competing demands on their time, being able to respond outside standard working hours may offer an advantage over more traditional forms of communication (Hutton et al., 2018).

Similarly, patients with chronic or long-term conditions are thought to benefit from access to digital communication when self-management advice and support can be made readily available. Young patients, in particular, are open and responsive to this type of support (Griffiths et al., 2018). Digital communication may also support and improve two-way communication between patients and their therapists, providing opportunities for provision of, and response to, personalized advice, information and support (Shaw et al., 2020).

Parents and carers of disabled children share many of the characteristics of patients who are believed to benefit from this type of communication with services. They have complex lives, juggling other competing responsibilities with caring for their disabled child who may require long-term support extending into adulthood (Contact a Family, 2017). Many parents and carers of disabled children are isolated, may not have access to sources of support (e.g. Baumgardner, 2019) and are living in poverty (e.g. Shaw et al., 2016). Online support may be especially helpful to mitigate these challenges (Baumgardner, 2019, op. cit.).

From a therapy perspective, supporting parents/carers is central to the success of interventions designed to promote the rehabilitation and participation of the child at home (Van Aswegen et al., 2019). As their child’s primary carer, they are required to manage their child’s day-to-day therapy needs at home – for example, use of specialized equipment – and to follow therapy advice related to feeding, toileting and dressing (Kruithof et al., 2020). Timely access to personalized information and advice related to their child and their child’s progress is essential (Novak and Berry, 2014). In this sense, the parent or carer has therapy support needs.

There is evidence in the UK to suggest that these therapy support needs are not always being met (Hutton et al., 2018). The provision and maintenance of specialist equipment to assist the child’s independence in daily living (e.g. seating; toilet etc.) was an area of concern; for example, provision of equipment that was inappropriate, of the wrong size, or uncomfortable. Delays in provision and lags in communication led to frustration and inconvenience for the parent and potential harm and discomfort for the child. Another concern related to difficulties experienced when contacting therapists using traditional means (e.g. messages left on the answerphone; failure of therapists to respond; lost messages). Therapy services are generally manned by small numbers of specialist therapists and parents found that it was often difficult to communicate with their nominated therapist (Hutton et al., 2018). This research reinforced our understanding of the support needs of parents/carers and raised the possibility that greater use of digital technology may be beneficial.

## Literature review

A review of the pre-covid literature found relatively little research on parents and carers of children with complex needs’ use of, and views on, digital communication technology in the care of their child, and contact with rehabilitation services. Blackburn and Read (2005) found that the majority of parents with a disabled child had used the internet to search for information relating to their child but there were a number of technical barriers to its use. Dehoff et al. (2016) highlighted the role of social media and apps in supporting parents of children diagnosed with special health care needs. Gardner et al. (2016) found a generally positive view of the potential for occupational therapy to be provided through digital technology in parents of a disabled son or daughter – though the need for more support and training for families was highlighted. Studies of other parent groups at this time – for example, parents of children with autism – suggested that they could find telehealth interventions convenient, collaborative and empowering (Wallisch et al., 2019) but that those with fewer internet skills and lower satisfaction with services were less likely to enrol in telehealth programmes (Salomone and Maurizio, 2017). However, we could find few studies specifically focused on the views of parents of children with complex needs and which explored this in the context of more general parent-led use of digital technology in the care of these children and as a support for these parents as carers, prior to the pandemic.

Pre-COVID19, the World Health Organization’s (2019) guidance for researchers recommended an evidence-based approach prior to developing any digital solutions to health care issues. The guidance emphasized the importance of evaluation of new methods alongside conventional approaches and considering their acceptability and feasibility to both patients and clinicians (WHO, 2019). The advent of COVID-19 created major disruptions in the delivery of rehabilitation services such as physiotherapy and occupational therapy (World Physiotherapy, 2021; Ganesan et al., 2021) and ‘forced’ services to quickly adopt online methods of delivery to parents of children with complex needs, as an emergency response (Ye, 2020), when these caregivers were experiencing high levels of stress and challenges to family wellbeing (Rakap et al., 2022). This occurred without the time to assess or evaluate best practice in this area or the impact on, or views of, parents and children using these services. Little was known about these parents and carers’ current use of digital technology in the care of their child or how acceptable this was to them.

Since the pandemic, there have been favourable reviews of the clinical effectiveness of some online paediatric services compared to face-to-face delivery across a range of disabilities (e.g. Dehghani et al., 2023; Ellison et al., 2021; Ogourtsova et al., 2023); studies of professionals’ views on the use of telemedicine with children with cerebral palsy and other neuromuscular complex chronic conditions (e.g. Nguyen et al., 2023; Wittmeier et al., 2022); and a small number of qualitative and mixed methods studies on family perspectives on and satisfaction with paediatric telehealth services. Some of these found that parents of disabled children were generally satisfied with these services (Tanner et al. 2020); held generally positive views and felt that they could enhance parent-provider communication and shared parental involvement (Smith et al., 2023). However, others have suggested some ambivalence, in that while parents found telehealth services useful, there were challenges from the lack of direct contact (Kloze and Wojtal, 2021) and parents would prefer them to be offered as an optional delivery mode in future to complement face-to-face delivery rather than to replace it (e.g. Portillo-Aceituno et al., 2022). Again, none of these explored parents’ wider use of technology in the care of their child.

## Aim

The aim of this study, which was undertaken prior to Covid-19, was to explore parents and carers’ confidence in using technology, frequency and reasons for use and attitudes to digital communication with services. It was based on previous extended consultation work with a group of parents/carers whose children with complex needs used rehabilitation therapy services and which explored research topics of importance to them (Hutton et al., 2018). During that consultation work, increasing the range of digital communication with rehabilitation therapy services was highlighted as a potentially beneficial area. For example, one parent spoke about how having video consultations would have helped resolve problems of being sent unsuitable equipment for their home. From this and other related work came the impetus for an exploratory study in this area.

We used a broad definition of technology in this survey to encompass the use of smartphones, tablets, computers, and other types of digital communication. This allowed respondents to interpret the questions and provide us with an insight into their different uses of technology in their role as carer, alongside their willingness to adopt new forms of communication with therapists. This study presents an insight into this use of digital communication, which has greater relevance in light of the wider adoption and continuing use of online methods since the pandemic. The study obtained ethical approval from Canterbury Christ Church University Ethics Committee (project number 17/H&W/34C).

## Methods

An online survey platform (Onlinesurveys.ac.uk) was used to design and distribute the questionnaire for parents and carers. A mix of open and closed questions focused on parents/carers’ perceptions of and use of digital technology – here defined as mobile phones, computers and tablets – in their role as a carer of at least one child who uses/used occupational therapy, speech and language therapy or physiotherapy services. We also included an adapted version of The Media and Technology Usage and Attitudes Scale (MTUAS, Rosen et al., 2013), a measure of confidence in use of technology – to assess positive and negative attitudes with items such as:

-  ‘I feel it is important to be able to access the Internet any time I want’
-  ‘I think it is important to keep up with the latest trends in technology’
-  ‘New technology makes people waste too much time’
-  ‘New technology makes people more isolated’

The first page of the survey provided information on the study followed by a series of questions that asked for consent. The main survey questions did not open until a participant had completed the consent questions and the survey was anonymous and did not ask for any names or contact details of respondents. Participants gave their consent by actively indicating this through the questions at the start of the survey and actively submitting their data at the end of the survey. As voluntary participants, they were informed that they had the right to withdraw consent at any point during the completion of the questionnaire and details were given of how to contact the research team should they wish to do this. Respondents were asked to create an anonymous, unique ID that they could provide to the researchers should they want to withdraw their responses at a later stage.

As an exploratory study, our aim was to gain a broad overview of parents and carers’ views. Therefore, a variety of routes to recruitment were used. These included distributing links to the survey via the Twitter feed of a children and family research centre at the authors’ home university, special schools in East Kent, UK, national rehabilitation professional networks, online parent-carer support forums and networks for parent/carers of children with disabilities and Mumsnet surveys nationally.

Descriptive statistical analysis was undertaken on the quantitative data in the survey. Technology confidence scores were calculated and a range of bivariate statistics, such as non-parametric correlations, chi squares and Mann–Whitney *U* tests were undertaken to explore group differences and for cross-tabulation of responses. The qualitative data from open questions was content analysed independently by the three researchers and emerging themes were then discussed as a group, compared and agreed.

## Results

### Overview of respondents

Responses were received from 43 parents or carers of a child or young person with a disability or additional needs. All respondents had at least one child who used/had used occupational therapy, speech and language therapy or physiotherapy. Respondents had between 1 and 6 children (total number of children across sample = 92). Each respondent had between 1 and 4 children who was/had been using one or more therapeutic services. Children were aged between 0 and 25 with more than two-thirds between 6 and 10 years (31 children) and 11 and 18 years (32 children).

Forty respondents (93%) were female, 3 (7%) were male. Respondents were aged from 26 to 65. All respondents spoke English as the main language at home/with their families except one, who spoke Tamil.

### Parents/carers’ current use of technology

All parents/carers responding said that they used a smartphone, tablet or computer in their role as a carer to their child. Table 1 shows the most popular reasons for use, selected from a provided list.

**Table 1.** Reasons for parents’ and carers’ use of smartphone, tablet or computer in their role as carer to a child with complex needs.

Reason for use	% (*n*)
To find information	95% (40)
To communicate with the services their child uses	88% (37)
To support their child’s therapy	81% (34)
For peer support from/with other parents	79% (33)

Open responses provided more detail on the reasons for use of smart phones, tablets and computers (Table 2).

**Table 2.** Reasons for parents/carers’ current use of technology (open responses).

Reason	Comments
Find information on services	‘[it is a] *Valuable way to find information . . .about the law to fight LA [local authority providing services] withholding funds and denying my son’s rights’* ‘[I use a] *Facebook group for rare conditions, researching equipment available, finding legal help when let down by services*’ *‘Information that allows us to negotiate what care and support our child is entitled to*’
Communication	*‘Day-to-day contact with my child’* *‘Virtual GP appointments using computer with webcam’* *‘Submit my carer’s hours and payments’*
Support (Child/ Therapy)	*‘Find information about my child’s condition’* *‘Essential to keep up-to-date with therapy methods’* *‘My child has a communication aid I can program this’* *‘Use of tablet to distract [child] when we are changing his tube/tape . . .and to encourage him to complete tasks he does not always enjoy’*
Support (Carer)	*‘Mindfulness app’* *‘Occasional use of twitter to make comments, often about good or lack of good facilities [in local area]’*

Facebook and Twitter were mentioned as a means of sharing experiences with other parents/carers by almost half the sample (19, 45%). Parents and carers also used their phones for reasons other than communication. They searched for information frequently on the internet, watched video clips or downloaded media files.

Parents and carers reported that alongside the use of smartphones, computers and tablets, they used other technology – ranging from sophisticated assistive technology such as an eye gaze device to audiobooks and game consoles.

### Frequency of use

Parents/carers were frequent users of technology as a means of communication – using their smartphones or tablets regularly to send and receive emails, texts and calls (Table 3).

**Table 3.** Frequency of use of smart phones, tablets or computers for different purposes.

Activity	Percent (n) reporting daily or more frequent activity
Texting	100% (43)
Using the internet	93% (39)
Checking voicemails	90%(38)
Using apps	90% (38)
Watching video clips and media files	85% (36)
Searching the internet for information	85% (36)
Reading, liking and commenting on social media	76% (32)
Taking pictures or recording videos	70% (29)
Making phone calls	62% (26)
Sending and receiving emails	56% (23)

### Attitudes to technology and the Media and Technology Usage and Attitudes Scale (MTUAS)

The questionnaire included questions taken from a subscale of a measure of attitudes to technology – the MTUAS, (Rosen et al, 2013). Parents/carers responded to the MTUAS questions in ways suggestive of holding broadly positive attitudes to technology. They wished to find information whenever they wanted online (95%, 41) and be able to access the internet at any time (86%, 37). They wanted to keep up with trends (77%, 33) and thought that tech could provide solutions to many of their problems (56%, 24).

The Cronbach’s alpha for the MTUAS data was 0.744 suggesting an acceptable level of inter-item consistency in the scale.

We found no closely comparable group data on MTUAS subscale scores from other studies.

To our knowledge, this was the first use of the MTUAS in a population of carers of children with complex needs (literature search and personal communication with author of scale).

### Potential of technology to play a greater role in supporting parents/carers of children with complex needs

The majority of respondents (91%, 39) thought digital technology could definitely or possibly play a bigger role in supporting them as a carer. Respondents also thought that technology could/might play a bigger role in supporting other parents as carers (88%, 38); and that technology could/might play a bigger role in supporting communication with services (84%, 36). Parents responded to open questions on the ways in which technology might play a bigger role in supporting parents. Responses were grouped into themes and are shown below (Table 4).

**Table 4.** How respondents thought technology could better support parents/carers of children with complex needs (open question).

Themes	How respondents thought technology could play a bigger role in supporting parents/carers
*Searching for Information*	• *‘Enable me to quickly source information that will make my family’s life more manageable’* • *‘More instant support /information. I waste a lot of time chasing people via others to get answer’* • *‘[digital technology] is a good way for parents to get information’*
*Support for parenting*	• *‘Videos and podcasts could help with strategies to help my daughter’* • *‘Tips and training on how to support your child would be great. For example, how to deal with meltdown in a child with ASD or sleep issues’*
*Support for parents and carers’ mental health and wellbeing*	• *‘Useful to access support as a carer, helpful to have support for the parent as well as child. Little support available if you are isolated impacting on mental health and wellbeing’* • *‘Could help manage my own mental health and stress’* • *‘[I] access several forums for parents asking for advice and contributing from experience’* • *‘Support from other parents online is a useful tool’* • *‘With some friends I have set up a Facebook group for SEN [Special Educational Needs] parents which has grown from 12 to over 1100 in four years. People feel isolated and need to know they aren’t alone in their challenges’* • *‘Just being in touch with each other, sharing S & L [speech and language] tips and advice’*
*Support for children’s learning, communication and participation*	• *‘Children could use [digital technology] to communicate with other children with SALT [Speech and Language Therapy] (sic) issues’* • *‘Tablets and phones assisted technology – a tablet could make a difference to child’s learning’* • *‘Technology to support the child such as apps and eye gaze technology – [there is] not enough of this’* • *‘SEN Kids are using the same technology as their friends. It means they have something in common rather than different’*
*Supplementing services /gaps in services.*	• *‘With lack of SALT provision an online resource would be helpful’* • *‘Emphasis should be on supporting service providers . . .if a SALT could see 10 patients virtually in a day versus 3 in person’*

### Changes to technology that would support parents and carers most

A closed question asked parents to say which types of changes to technology would help them most. Table 5 shows that improved and more relevant content was the change seen as most useful.

**Table 5.** How technology could play a greater role in supporting parents/carers of children with complex needs (closed question).

	Percent (n)
Improved/more relevant material/content	72% (31)
Training/help in different forms/applications of technology	51% (22)
More user friendly/easier to use technology	49% (21)
More information on where/how to find/access technology	47% (2)
Support with the costs of accessing and using technology	40% (17)
Access to a smartphone, tablet or computer	28% (12)

Respondents detailed a number of important ways in which they felt greater and better use of technology to communicate between services and families could be beneficial (Table 6).

**Table 6.** Ways technology could be used to provide more benefits to children and parents/carers (free text comments).

*‘To show/remind you what your child’s therapy plan is and how they are doing’*
*‘Online resource or apps would enable us to provide some therapies ourselves’*
*‘Parents can feel empowered when advice is shared via an email, improves their day/week without a face-to-face appointment’*
*‘Daily/weekly updates via web on therapies tried at home would be useful for both sides’*
*‘I think a central communication system . . . accessible by all professionals involved . . .would be ideal’*
*‘Quicker access to therapists especially for minor worries’*
*‘[Skype appointments]‘Saves time attending meetings. EHCP[Child’s Education and Healthcare Plan which details services and support provided] could be refined via email and editable attachments’*
*‘Text reminders and warnings if clinics are running late – option to face time or SKYPE professionals’*
*‘Email has been brilliant especially when accessing OT [Occupational Therapy] and SALT’*

### Potential drawbacks of, concerns about, and barriers to, greater use of technology in parent/carer-services communication

Despite the many perceived benefits of greater use of technology among this population, a number of concerns, issues and barriers were raised. Some respondents reported that greater electronic communication should not occur at the expense of the availability of face-to-face contact and should complement this rather than be used to replace it. Data protection was also a concern for some respondents, along with structural and policy-based issues (Table 7).

**Table 7.** Concerns around, and barriers to, greater use of technology to support parents/carers.

*‘Cost-cutting with technology-based interactions rather than face to face will not help parents, especially when interaction with therapists is already poor’*
*‘Services are stretched to breaking point so getting replies is already next to impossible. Would technology help or could it lead to further cuts?’*
*‘Security/data protection can be an issue and stops sharing but sensible use of tech gets round this’*
*‘The technology is there but communication problems arise because of policies and lack of funding to use it properly’*
*‘A frequent reaction [from professionals]. . .is that it is not possible to communicate via email. All sorts of spurious reasons are given . . .and it takes a highly informed parent to make the case for use of technology for communication. It’s frustrating and time wasting that services do not use a range of tech for communicating and rely on hard copy in the post or phone . . .which are profoundly difficult for working parents.’*
*‘My own knowledge and confidence are limited in this field – I’m self-taught but seem to find what I need most of the time’*
*‘Not everybody wishes to use technology due to cognitive ability, online security, financial reasons’*

## Discussion

Previous research has highlighted the importance of the internet as a source of information for families since the start of the World Wide Web in the early 1990s (Lupton et al., 2016). The internet provides families with disabled or sick children the means to access both information and also valuable support from other parents with similar experiences (Blackburn and Read, 2005; Plantin and Daneback, 2009). The aim of this study was to gain greater understanding of parents and carers’ attitudes to and use of smartphones, laptops and computers – in their role as a carer.

Despite being a small sample of parents/carers, most participants provided detailed responses to open questions providing valuable insights into their experiences. The findings from this survey support previous research about parents/carers’ use of this technology, while providing new insights into parents’ attitudes in this area.

The results reflected levels of ownership and use of digital technology amongst the general UK population (e.g. Ofcom, 2018) with all participants reporting that they owned a smartphone, tablet or computer and used this technology frequently in their role as a carer. Almost all parents and carers (95%) were using digital technology to search for information about their child’s condition, and the majority were also using it to communicate with services, support their child’s therapy and seek support from other parents.

Digital technology served a range of purposes for parents and carers. Most frequent of these was searching for specific information about their child’s condition. This was mentioned by 95% of parents/carers responding to this survey-confirming findings from a systematic review of health-related internet use by informal carers of children and adolescents that found that carers accessed disease-specific information to assist them when making decisions about treatment for their child (Park et al., 2016); next most frequent uses of technology were communicating with services (88%) and supporting their child’s therapy (81%).

Most participants (79%) also reported using online media channels such as Twitter and Facebook specifically to share advice and address feelings of isolation. As noted by others, the internet can help parents of disabled children overcome the sense of social isolation that many experience (Newman et al., 2019). Social support via virtual communities and email have previously been demonstrated as being useful for informal caregiver emotional support (Park et al., 2016). However, a scoping study by Gruebner et al. (2022) highlighted the continuing need for better understanding of parents’ use of digital platforms for this purpose, their effectiveness and ethical and privacy concerns.

Respondents also highlighted the value of being able to source relevant information quickly as a way of keeping up-to-date with developments. Several parents mentioned the use of the internet to inform themselves about their child’s rights and entitlement to services. This possibly reflects the high levels of dissatisfaction amongst parents of children with additional educational needs about the lack of resources available to support implementation of Education Health and Care Plans (Ofsted, 2020).

As well as exploring parents/carers’ reasons for using digital technology, the study found some specific types of digital activity that were particularly well used. For example, respondents reported texting at least daily. This corresponds with other research, and has been successfully capitalized on for a number of years, with texts to remind patients of appointments across a number of services in the US and Europe (e.g. Dash et al., 2016; Schwebel and Larimer, 2018) – though this is less common in rehabilitation services. There may be potential for greater use of texting to support parents and carers of children using these services. The results also suggest that as well as using technology for communication, parents and carers use it to view videos and download media files. This could create possibilities in terms of exploring this as a means of providing information to parents, as an alternative to leaflets and paper-based information.

The majority of respondents (91%) felt that digital technology in the form of smartphones, tablets and laptops could play a greater role in supporting them in their role as carers, as well as supporting other parent carers (88%). Communication with services was the main area in which they thought it could do this (84%). The open responses provided us with a rich and varied range of ideas about how parents/carers thought that technology could be used to best effect. This included ideas such as provision of remote (online) support from therapists. Parents were also concerned about losing the personal contact that they had with therapists. The optimum use of the technology would be to enhance and not replace the existing services. This corresponds with post-covid research on parents’ views of digital early intervention services for children with disabilities (e.g. Portillo-Aceituno et al., 2022).

At the time of the study (pre-COVID-19), there were few examples of remote support provided to parents of disabled children. Where this approach had been adopted had been in areas where geographical constraints made it difficult to provide services, for example, Australia (Parsons et al., 2017), and where there was a recognized shortage of specialist support available. Since the pandemic, online rehabilitation has rapidly emerged as an emergency response to delivering services (e.g. in South Africa: Khatib and Hlayisi, 2022; Marsden and Docherty, 2021) and its advantages and a desire not to ‘go back to normal’ has been expressed by some professionals (Rosenbaum et al., 2021). While there is a growing evidence base around its clinical effectiveness for this population, there remains minimal in-depth research on parental perspectives and less still focused on children’s voices. With the need to capitalize on the best use of digital technology in the continued care of children with disabilities and in preparing for the possibility of future pandemics, more detailed work on the impacts on and views of the family and their use of digital technology more broadly remain important areas for policy and practice and research.

### Limitations

There were a number of limitations to this study. The sample was small and cross-sectional and as data were collected via an online survey it was likely to be skewed towards parents with greater confidence in and frequency of use of the digital technology being explored. Larger, more deliberatively representative and longitudinal data would be beneficial and future studies in this area might address this.

For simplicity, we used a very broad definition of technology. Respondents’ answers might change dependent on the specific type of technology in question and this is an area that could be explored in more detail in future studies.

We chose not to ask parents to disclose information about the specific nature of their child’s disability, instead focusing on the regular use of two or more rehabilitation services. Future studies with larger samples might gather more detailed information in this area – if it was felt to be beneficial for more detailed subgroup analysis.

**Key findings**

• Parents/carers of children with complex needs, consulted prior to COVID-19, were active users of, and held positive attitudes towards greater use of, digital technology in their caring role. There was a desire to see online service delivery supplement and not replace face-to-face services. This corresponds with findings from other post-covid research.

**What the study has added**

This study addresses the gap in our knowledge about attitudes towards, and use of, technology by parents/carers of children using rehabilitation services in their caring role, pre COVID-19. It highlights the role of digital technology in family care for children with complex needs; and the preference for hybrid service delivery, where digital delivery and communication modes complement rather than displace in-person contact with professionals.

It is recommended that, where possible, services should offer flexible delivery modes to meet the preferences of families. Further longitudinal research on the role of digital technology in the family-based care of children with complex needs is recommended.
